# Dual Inhibition of Pirarubicin-Induced *AKT* and *ERK* Activations by Phenformin Sensitively Suppresses Bladder Cancer Growth

**DOI:** 10.3389/fphar.2019.01159

**Published:** 2019-10-08

**Authors:** Mei Peng, Jun Deng, Sichun Zhou, Di Xiao, Jiahui Long, Nan Zhang, Caimei He, Miao Mo, Xiaoping Yang

**Affiliations:** ^1^Departments of Pharmacy and Urology, Xiangya Hospital, Central South University, Changsha, China; ^2^Key Laboratory of Study and Discovery of Small Targeted Molecules of Hunan Province, Department of Pharmacy, School of Medicine, Hunan Normal University, Changsha, China

**Keywords:** *Akt*, *ERK*, pirarubicin, phenformin, bladder cancer

## Abstract

Activations of Akt or ERK pathway induced by clinical drugs promote therapeutic failure due to decrease of drug response, and no available strategies have been developed to solve these problems. In this study, we found that pirarubicin (THP), one important chemotherapeutic drug for treating bladder cancer intravesically, dramatically elevated phosphorylations of both Akt and Erk1/2 in addition to inducing DNA damage. MK2206 or AZD6244, representative Akt and Erk1/2 inhibitors, respectively, profoundly sensitized bladder cancer cells to THP treatment. Interestingly, we found that inhibition of a single arm of either Akt or Erk1/2 pathway would induce the increase of another arm, indicating the existence of the crosstalk between these two pathways. Thus, simultaneous suppression of both signals may be needed for increasing the sensitivity of THP. On the other hand, we revealed that phenformin efficiently inhibited both Akt and Erk1/2 phosphorylation in a dose-dependent manner. Furthermore, we demonstrated that phenformin, mimicking dual inhibitors, plays dramatically synergistic action with THP both *in vitro* and *in vivo*. Our findings suggest that combination therapy of THP with dual inhibitors may constitute a successful strategy for improving chemotherapy response.

## Introduction

Bladder cancer is one of the most frequent cancers of the urinary tract; it accounted for about 60,490 new cases and 12,240 deaths in the United States in 2017 (Siegel et al., 2017). Approximately 75% of patients with urothelial carcinoma of the bladder initially present with non–muscle-invasive disease and transurethral resection (TUR) has served as the standard treatment in the clinic (Clark et al., 2013; Babjuk et al., 2017). Unfortunately, up to half of patients tend to recur or invade to higher grade (van Rhijn et al., 2009; Chang, 2017). Therefore, prophylaxis of the high frequency of recurrence after TUR is important.

It is reported that Akt and ERK signaling are dysregulated or mutated in human cancers, including bladder cancer (Koboldt et al., 2012; Calderaro et al., 2014; Tan et al., 2019). These genetic mutations have been validated as an essential step in the initiation and progression of human tumors. Downregulation of phosphorylated Akt or ERK levels has been demonstrated to induce cell cycle arrest and apoptosis (Balakrishnan et al., 2015; Brown et al., 2015; Hayes et al., 2016; Zeng et al., 2016). On the other hand, the hyperactivations of Akt and ERK pathways, as a consequence of diverse clinical cancer therapies (chemotherapy, targeted therapy, and radiation therapy), play an important role in drug resistance (Obenauf et al., 2015). The commonly clinical used chemotherapeutic drugs such as doxorubicin and paclitaxel have been found to be resistant accompanying with upregulation of pAkt (Hirai et al., 2010). In BRAF-driven melanoma, Akt activation was observed in the clinic during vemurafenib treatment. This therapy-induced activation not only enhances the survival of drug-sensitive cells, but also acutely accelerates the expansion and dissemination of drug resistance (Mao et al., 2013). In BRAF-mutant colorectal cancer, sustained ERK pathway activation has been demonstrated to confer resistance to RAF/EGFR or RAF/MEK combinations (Ahronian et al., 2015). Aforementioned studies suggest that suppression on Akt or ERK may be a candidate strategy to increase treatment response and improve clinical cancer therapeutic efficacy. Nevertheless, the effect of a single inhibition may be weakened due to the crosstalk between these two signaling pathways (Mendoza et al., 2011; Sathe et al., 2014; Kashyap et al., 2018). Thus, simultaneously inhibiting both Akt and ERK pathways rather than a single pathway would be a more efficient approach to ameliorate drug response and suppress tumor growth (He et al., 2019; Yu et al., 2019).

Pirarubicin (THP), one representative compound of anthracyclines, prevents protein synthesis through binding DNA base pairs and inhibiting topoisomerase II, which shows a greater antitumor activity with better local toxicity profiles than its analog doxorubicin (Dantchev et al., 1979; Tsuruo et al., 1982). Consistent with the guidelines of the European Association of Urology on non–muscle-invasive bladder cancer, immediate instillation of THP after TURBT has been demonstrated to significantly reduce the risk of 1-, 2-, 3-, and 5-year recurrence rates, especially in low- and intermediate-risk non–muscle-invasive bladder cancer patients in Chinese and Japanese patients (Stewart and Mostafid, 2005; Ito et al., 2013; Kang et al., 2016). Unfortunately, the 5-year recurrence rates are greater than 50%, especially higher in patients with EORTC scoring greater than 5 (Li et al., 2013; Ding et al., 2018). In addition, a chemosensitivity test for human genitourinary tumors showed unexpectedly low response rate of THP (19.7%) treatment (Koshida et al., 2005). Thus, enhancing THP response holds a great promise for its clinical application.

Recently, metformin, one of typical biguanide compounds, has shown its multiple functions including antiaging and anticancer properties except its excellent antidiabetes activity (Podhorecka et al., 2017; Saini and Yang, 2018; Piskovatska et al., 2019). In previous study, we demonstrated that metformin decreased phosphorylation of both Akt and Erk1/2 to inhibit bladder cancer (Peng et al., 2016). However, its drawbacks, particularly, unavailable high concentration on targeting tumor locations for its anticancer activity, have been reported (Menendez et al., 2014; Chandel et al., 2016). Studies including another recent work in our laboratory have shown that phenformin, another biguanide compound, has a much stronger anticancer activity than metformin (Huang et al., 2018). However, it remains unknown whether its action was accompanied by inhibiting phosphorylated Akt and ERK.

In the present study, we first explore THP’s effects on the protein levels of pAkt and pErk1/2. Then we explore whether phenformin could inhibit both Akt and ERK phosphorylation. Finally, we investigate whether phenformin could enhance THP sensitivity. We expect that combination of phenformin with THP could be a novel strategy to suppress bladder cancer growth for future clinical administration.

## Materials and Methods

### Reagents

Phenformin (Aladdin Chemistry, Shanghai, China) was prepared in a range of concentrations in culture medium. Pirarubicin, AZD6244, and MK2206 (Selleck-Biotool, Shanghai, China) were prepared as a stock solution of 50 mM in dimethyl sulfoxide (DMSO).

Antibodies against the following target proteins were obtained from Cell Signaling Technology (Beverly, MA, USA): phospho-p70 S6 kinase (Thr389), total Akt, phospho-Akt, total mammalian target of rapamycin (mTOR), phospho-mTOR (Ser2448), total 4E-BP1, phospho-4EBP1, total Erk1/2, phospho-Erk1/2, phosphor-H2A.X, and β-actin.

### Cell Lines and Culture Conditions

The murine and human bladder cancer cell lines MB49 and UMUC3 were generously provided by Dr. P. Guo of the Institute of Urology at Xi’an Jiao tong University (Xi’an, Shanxi, China). All cell lines were cultured in Dulbecco’s modified eagle medium (Hyclone, Logan, UT, USA) supplemented with 10% fetal bovine serum (FBS; Hyclone) and 1% penicillin–streptomycin. Cultures were incubated at 37°C in humidified air containing 5% CO_2_.

### Cell Viability Assay

Cell viability was assessed using a tetrazolium-based assay. Briefly, cells were seeded at 8 × 10^3^ per well in 96-well culture plates and incubated in medium containing 10% FBS. At 24 h later, cells were treated for 48 h with different drugs. The tetrazolium salt of MTT (50 µl; Sigma) was dissolved in Hanks balanced salt solution to a concentration of 2 mg/ml and added to each well. The plates were incubated for another 5 h. The medium was aspirated from each well, DMSO (150 µl; Sigma) was added to dissolve formazan crystals, and absorbance was measured using a microplate reader (Synergy HTX; Biotek, VT, USA) at 490 nm (against reference absorbance at 630 nm). Dose–response curves were generated and used to calculate the half-maximal inhibitory concentration (IC_50_) using SPSS 16.0 (IBM, Chicago, IL, USA).

### Clonogenic Assay

Briefly, 8 × 10^3^ cells were seeded into 24-well dishes in 0.5 ml of medium. At 24 h, cells were treated with drugs for a further 6- to 8-day period in medium containing 10% FBS. Cells were fixed with 10% formaldehyde and stained with 0.1% crystal violet. Absorbance was measured using a microplate reader (Biotek) at 550-nm wavelength.

### Apoptosis

Apoptosis was assessed using flow cytometry in a separate experiment. Briefly, cells treated with different drugs were harvested with trypsinization, washed twice with phosphate-buffered saline (PBS), and resuspended in 1× binding buffer to 1 × 10^6^ cells/ml. One hundred microliters of the solution (1 × 10^5^ cells) was transferred to a 5-ml culture tube. Five microliters of fluorescein isothiocyanate (FITC)–annexin V and 5 µl propidium iodide (PI) were added. The cells were gently vortexed and incubated for 15 min at room temperature (25°C) in the dark. Four hundred microliters of 1× binding buffer was added to each tube. Analyzed by flow cytometry within 1 h, labeled cells were counted by flow cytometry on a FACS Calibur flow cytometer [excitation wavelength, 488 nm; emission wavelengths, 530 nm (FL-1 channel, FITC) and 670 nm (FL-3 c3 channel, PI)]. Data were analyzed using Cell Quest software (Becton–Dickinson). Nonapoptotic cells were defined as those negative for annexin V and PI; necrotic/late apoptotic cells as those positive for both labels; and early apoptotic cells as those positive for annexin V but negative for PI.

### Western Blotting

Tissues and cell proteins were fractionated by sodium dodecyl sulfate–polyacrylamide gel electrophoresis, transferred to membranes, and then incubated overnight at 4°C with different primary antibodies described in Reagents section above (Cell Signaling Technology) in buffer containing bovine serum albumin. Membranes were washed with triethanolamine buffered saline (TBS) containing 0.05% Tween-20, blotted with secondary antibody for 1 h at room temperature, and then washed again three times. Pierce Super Signal chemiluminescent substrate (Rockford, IL, USA) was added, and the blot was imaged immediately on a ChemiDoc system (Bio-Rad, Hercules, CA, USA) and a Perfection V500 camera (Epson). Band intensities were quantified using ImageJ.

### Luciferase Transfection of MB49 Cells

The MB49 cells were infected with a lentivirus containing the firefly luciferase gene (ViGene Biosciences, Shandong, China). Briefly, MB49 cells were counted and seeded in six-well plates at a density of 1 × 105 cells/ml; 24 h later, they were infected with Lenti-luc virus (12 µl viral supernatant/ml medium) according to the manufacturer’s instructions.

### Orthotopic Implantation and Intravesical Treatment

Female C57BL/6 mice were purchased from Hunan SJA Laboratory Animal Co., Ltd (Changsha, Hunan, China). All our animal experiments were conducted in accordance with guidelines approved by the Institutional Animal Care and Use Committee at Hunan Normal University (Protocol 201703230). Exponential growth of MB49^luc^ cells (transfected with luciferase) (Zhu et al., 2014) was harvested, and cell density in collection tube was counted by cell counter. Female mice 6 to 8 weeks of age were used for cancer cell implantation. Briefly, 1.2 × 10^5^ MB49^luc^ cells in 0.1 ml PBS were injected into the bladder wall using 1 ml syringes and catheter scratching according to the previously described protocol (Yang et al., 2013). A total of five groups designed are normal female C57BL/6 mice, which received an instillation of 50 µl of PBS intravesically as control group without tumor; female C57BL/6 mice with orthotopic bladder cancer were randomly divided into four groups, which received an instillation of 50 µl PBS, phenformin (1.45 mg/ml), THP (0.25 mg/ml), or combinations of phenformin with THP (phenformin 1.45 mg/ml, THP 0.25 mg/ml) intravesically. Each group has 12 female C57BL/6 mice. All treatments started at day 2 post–tumor implantation, twice *per* week, and continued for 2 weeks. Tumor burden was checked weekly through Xenogen IVIS (*In Vivo* Imaging System) (PerkinElmer, Waltham, MA, USA). Mice were injected with Luciferin approximately 15 min prior to imaging.

### Immunohistochemistry

Tissue processing, pAkt, pErk1/2, p-mTOR, and hematoxylin-eosin staining of 6-µm tissue sections were conducted by the Department of Pathology, Xiangya Hospital, Changsha, Hunan, People’s Republic of China. The slides were reviewed by a pathologist (Dr. Jun Zhou).

### Statistical Analyses

All data are presented as mean ± SD. Statistical analysis was performed using SPSS 16.0 (IBM, New York, USA). Differences between groups were assessed for significance using Student *t* test for experiments involving only two groups and using analysis of variance and the least significant difference test for experiments involving more than two groups. Graphs were generated using GraphPad Prism 6.0. Two levels of statistical significance were considered: *P < 0.05 and **P < 0.01.

## Results

### THP Activates Akt and ERK in Addition to Inducing DNA Damage in Bladder Cancer Cells

**Figure 1:**
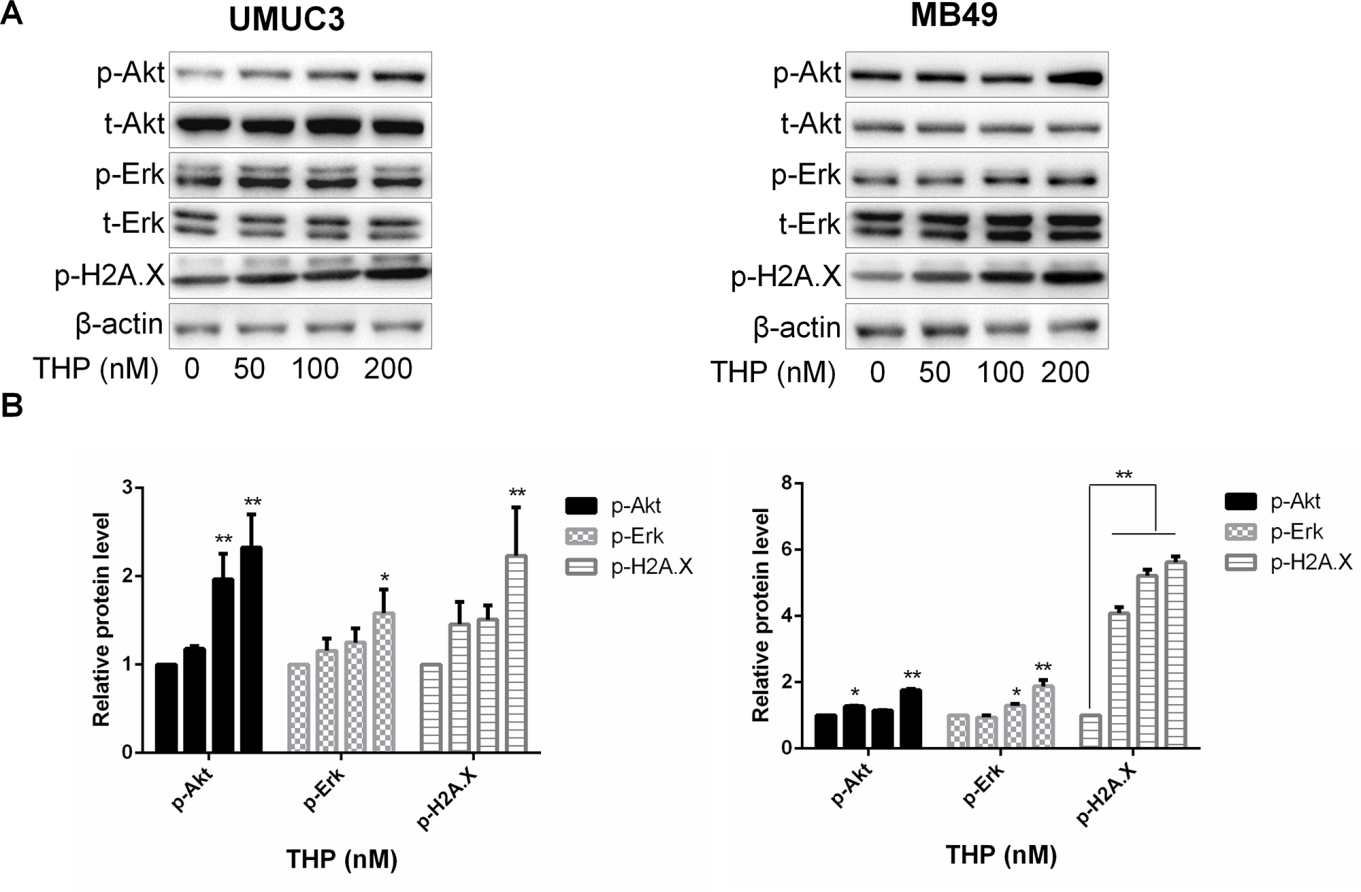
THP activates Akt and ERK in addition to inducing DNA damage in bladder cancer cells. **(A)** UMUC3 and MB49 cells were treated with an increasing concentration of THP for 36 h. Western blot analysis was used to examine total (t) and phosphorylated (p) forms of Akt and Erk, p-H2A.X. β-Actin was included as a loading control. **(B)** Relative levels of phosphorylated Akt, Erk, and H2A.X are shown as means ± SD, n = 3, **P < 0.01, *P < 0.05.

THP, a derivative of doxorubicin, exerts its anticancer effect through inhibiting DNA damage– and genomic instability–associated DNA topoisomerase II, which is critical for the proper functioning of DNA. Thus, we first examined the effect of THP on histone H2A.X, a marker of DNA damage and genomic instability. As expected, THP significantly enhanced phosphorylation of histone H2A.X, confirming its DNA damage effect in these two bladder cancer cell line models. It has been demonstrated that Akt and ERK signalings are closely connected with cell survival, promoting cell proliferation through interacting with kinases substrates or inhibiting tumor suppressor genes (Kashyap et al., 2018). To determine whether THP treatment induces alterations of either Akt or/and Erk1/2 phosphorylation, the levels of Akt and Erk1/2 phosphorylation were measured by Western blot in the THP-treated and control groups. Unexpectedly, exposure of murine and human bladder cancer cells to THP resulted in elevated Akt and Erk1/2 phosphorylation in the THP group compared with the control group (**Figure 1**). Therefore, these results confirmed that THP upregulates Akt and ERK phosphorylation except causing DNA damage, possibly initiating cell survival signal.

### Either Akt or ERK Inhibitor Sensitizes Cells to THP-Induced Death

**Figure 2:**
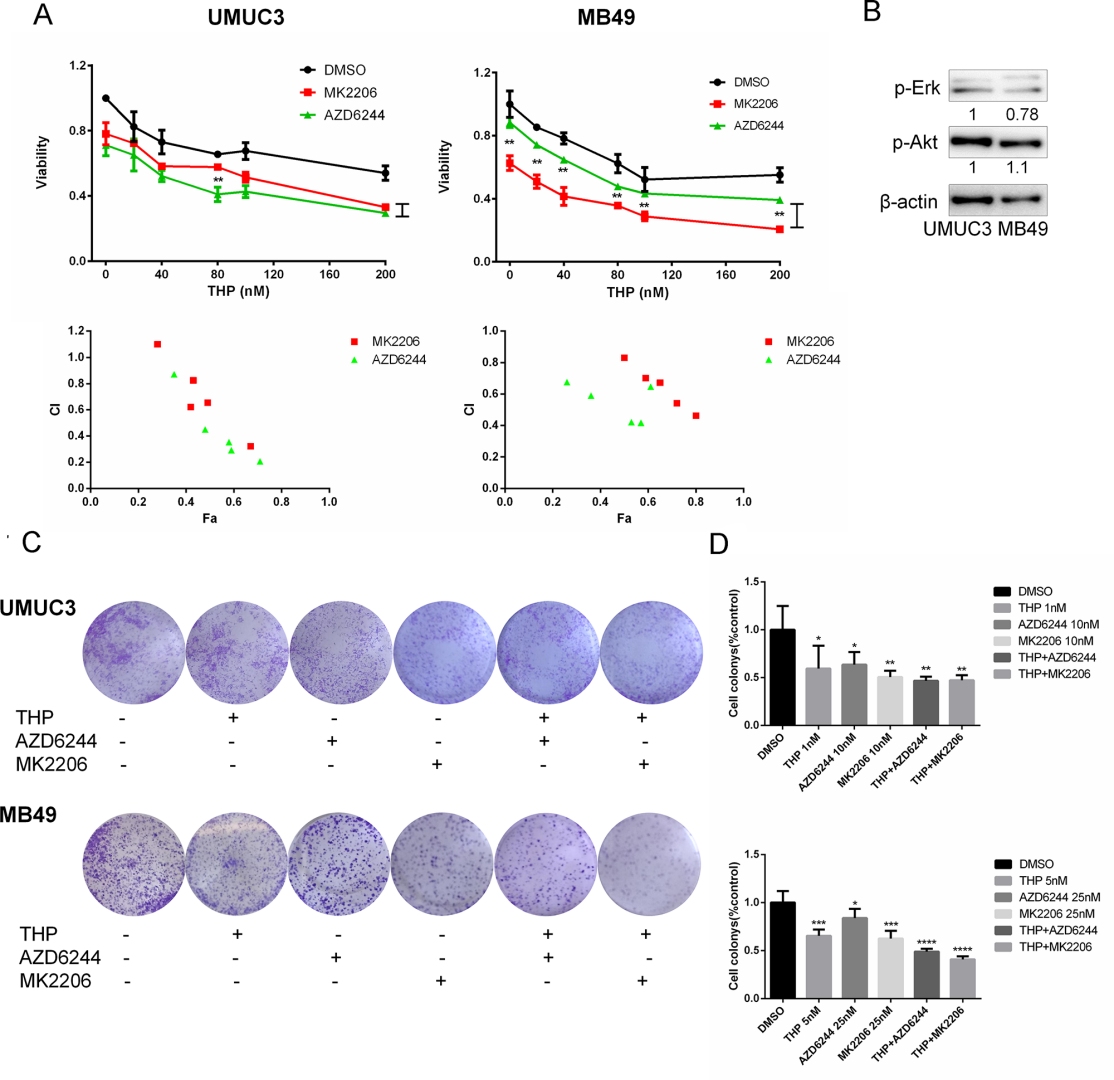
Either Akt or ERK inhibitor sensitizes cells to THP-induced death. **(A)** UMUC3 and MB49 cells were pretreated with 0.2 µM AZD6244 or 0.2 µM MK2206 for 24 h before exposure to increasing concentrations of THP for an additional 48 h. Cell viability is expressed as a percentage of viability observed in untreated cells. Below: Combination index (CI) among the combinations of two drugs was calculated using CompuSyn software. If CI = 1, it denotes additives; if CI >1, it denotes antagonism; if CI <1, it denotes synergism. CI values in the vast majority of combinations were less than 1, indicating synergism. Results are presented as the median of five independent experiments. Means ± SD, **P < 0.01, *P < 0.05. **(B)** The protein levels of pAkt, pErk in these two cell lines were detected by Western blot with β-actin as a control. **(C)** Evaluation of colony suppression by THP combined with AZD6244 or MK2206. UMUC3 and MB49 cells were treated for 7 days with THP combined with AZD6244 or MK2206 and then stained with crystal violet to allow colony counting. **(D)** Quantification of the experiments conducted in panels. Wells were scanned at a wavelength of 550 nm. Results are the mean ± SD of five independent experiments, ***/****P < 0.001, **P < 0.01, *P < 0.05.

Then we tested the hypothesis that inhibition of either pAkt or pERK using specific molecular inhibitors may improve THP efficacy. To accomplish this examination, we determined the combination of either MK2206 (Akt specific inhibitor) or AZD6244 (ERK specific inhibitor) with THP on cell proliferation. As shown in **Figure 2A**, single-agent THP limits the viability of bladder cancer cells in a concentration-dependent manner. However, the inhibitory percentage was less than 50% with 200 nM THP-treated alone. Then, pretreating cells with 0.2 µM MK2206 or AZD6244, the inhibitory effects were dramatically enhanced. The inhibitory percentage ascended to 70% when either MK2206 or AZD6244 was combined with THP at the concentration of 200 nM. Furthermore, there were significant differences between THP + AZD6244 and THP + MK2206 groups at all detected concentrations in MB49 cells. In UMUC3 cells, there was statistical significance between these two groups when the concentration of THP was 80 nM as well (**Figure 2A**). The majority of combination indexes (CIs) were less than 1, confirming the synergism between these two combinations, respectively. The above data indicated that inhibition of either Akt or Erk1/2 signals enhanced the antiproliferation effect of THP. We also observed an interesting phenomenon that AZD6244 pretreatment resulted in a more significant cell growth inhibition in UMUC3 than that in MB49 cells (*P* = 0.0147), while MK2206 pretreatment resulted in a more significant cell growth inhibition in MB49 than that in UMUC3 cells (*P* = 0.0305). We determined the protein levels in these two cell lines and found that pAkt in MB49 cells is higher than that in UMUC3 cells, while pErk1/2 in UMUC3 cells is higher than that in MB49 cells (**Figure 2B**). This phenomenon may be associated with the different antiproliferation efficacy of these two inhibitors in these cell lines due to the difference of pAkt and pErk1/2. However, we would like to point out that these differences may also stem from different species (human and mice). Colony formation was also examined to evaluate this combinational effect. As shown in **Figure 2C**, THP, AZD6244, or MK2206 treated alone markedly reduced bladder cancer cells colony formation ability with 40% inhibitory percentage in the 1 nM THP group, 36% in the 10 nM AZD6244 group, and 50% in the 10 nM MK2206 group. This reduction was further exacerbated while combining THP with either one of these specific inhibitors (**Figures 2C, D**). Taken together, these data revealed that combination of either Akt- or Erk1/2-specific inhibitors with THP exhibited remarkably synergistic anticancer effects; differences may also stem from different species (human and mice). Colony formation was also examined to evaluate this combinational effect. As shown in **Figure 2C**, THP, AZD6244, or MK2206 treated alone markedly reduced bladder cancer cells colony formation ability with 40% inhibitory percentage in the 1 nM THP group, 36% in the 10 nM AZD6244 group, and 50% in the 10 nM MK2206 group. This reduction was further exacerbated while combining THP with either one of these specific inhibitors (**Figures 2C, D**). Taken together, these data revealed that combination of either Akt- or Erk1/2-specific inhibitors with THP exhibited remarkably synergistic anticancer effects.

### Crosstalk Between pAkt and pErk1/2 in Bladder Cancer Cells

**Figure 3:**
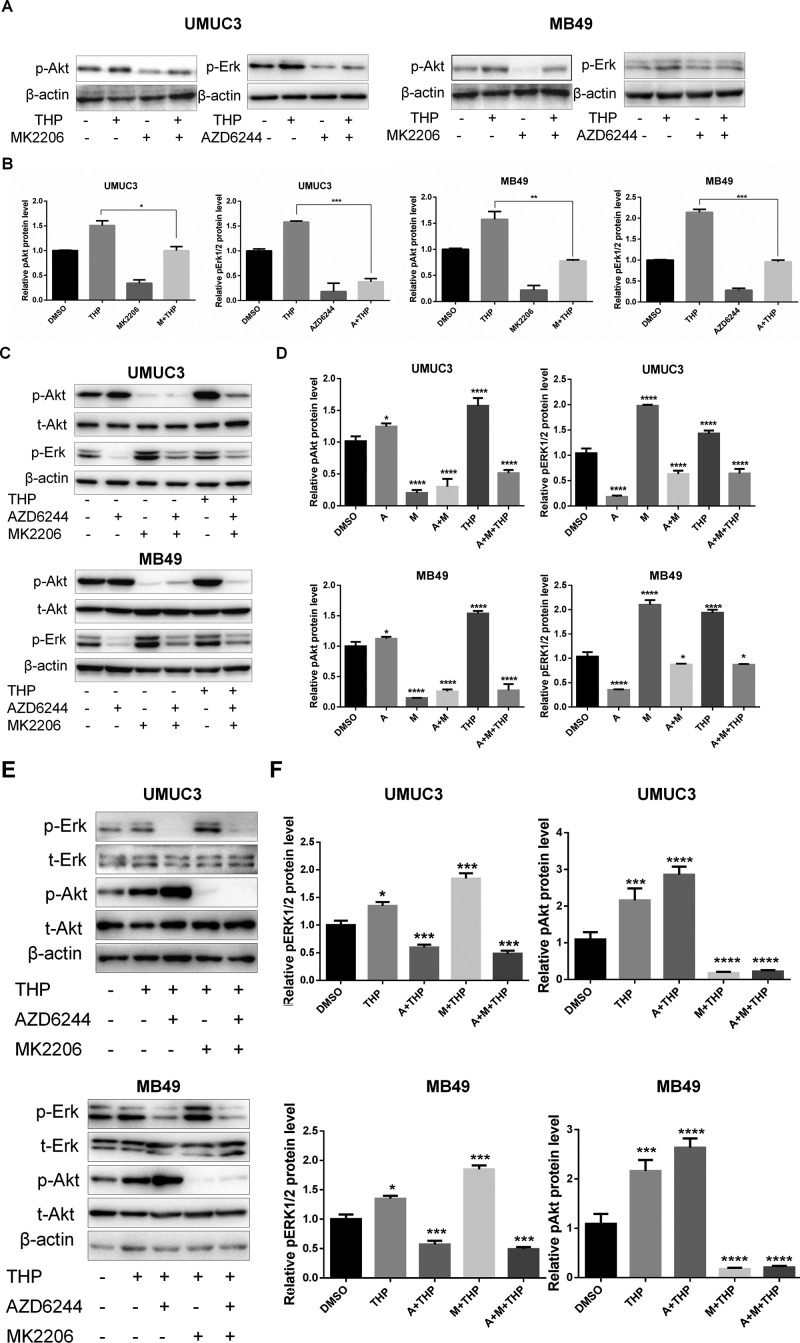
Crosstalk between pAkt and pErk1/2 in bladder cancer cells. **(A**, **C**, and **E)** UMUC3 and MB49 cells were serum-starved and pretreated with 0.5 µM AZD6244 or 0.5 µM MK2206 for 30 min before exposure to 400 nM THP for 10 h, and the protein levels of pErk, pAkt, and t-Akt were detected by Western blot with β-actin as a control. **(B**, **D**, and **F)** Relative levels of phosphorylated Akt and Erk (A: AZD6244, M: MK2206) are shown as means ± SD, ***/****P < 0.001, **P < 0.01, *P < 0.05.

Synergistic effects have been observed between combination of THP with MK2206/AZD6244. However, it has been demonstrated that crosstalk exists between the pAkt and pERK1/2 signaling pathways, which means that when one pathway is suppressed, the other will be enhanced to compensate for the weakened function (Hirai et al., 2010). Thus, the protein level alterations of pAkt and pErk1/2 after either MK2206 or AZD6244 incubation in both MB49 and UMUC3 cells were investigated. The results showed that MK2206 alone dramatically suppressed pAkt and alleviated the increase of pAkt induced by THP treatment as we expected. The similar phenomenon was observed on pErk1/2 when exposed to AZD6244 (**Figures 3A, B**). Nevertheless, we noticed that the level of pErk1/2 was upregulated in these two cell lines treated with MK2206, while the level of pAkt was elevated in cells treated with AZD6244 (**Figures 3C, D**). And these increases were further amplified when combining with THP (**Figures 3E, F**). Therefore, the potential crosstalk between pAkt and pErk1/2 inspired us to explore whether simultaneous inhibition on both may reduce their expressions. As shown in **Figures 3E and F**, when both MK2206 and AZD 62444 were applied, we did find that the expressions of both pAkt and pErk1/2 were markedly decreased, compared with single application of these inhibitors.

### Phenformin Substantially Reduces the Protein Levels of Both pAkt and pErk1/2 at Micromolar Concentration

**Figure 4:**
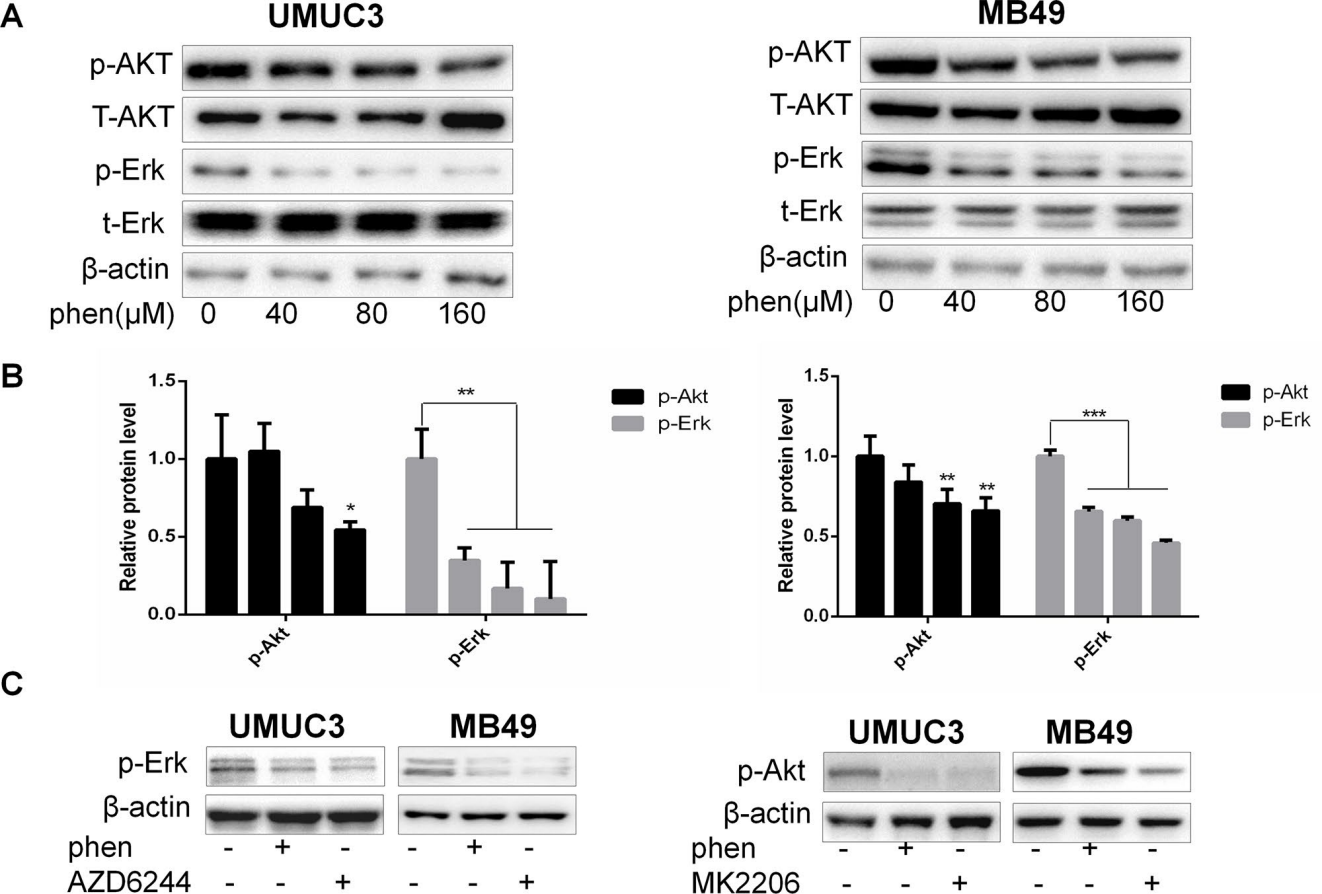
Phenformin substantially reduces the protein levels of both pAkt and pErk1/2 at micromolar concentration. **(A)** UMUC3 and MB49 cells were treated with an increasing concentration of phenformin for 36 h. Western blot analysis was used to examine total (t) and phosphorylated (p) forms of Akt and Erk. β-Actin was included as a loading control. **(B)** Relative levels of phosphorylated Akt and Erk. Results are mean ± SD. *P < 0.05, **P < 0.01, ***P < 0.001 vs. control. **(C)** UMUC3 and MB49 cells were treated with 200 µM phenformin, 0.5 µM AZD6244, or 0.5 µM MK2206 for 36 h, and the protein levels of pAkt, pErk were detected by Western blot with β-actin as a control.

Based on above results, it will be more powerful to enhance THP antitumor effects when combined with dual inhibition of pAkt and pErk1/2. Thus, it is meaningful to find compounds that can downregulate both pAkt and pErk1/2. We have reported that metformin exerts its anticancer property *via* decreasing phosphorylation of both Akt and Erk1/2 in bladder cancer. However, higher than 1-mM dose is needed for exerting sufficient efficiency (Peng et al., 2016). Reaching this dose in the clinic is impossible *via* conventional oral administration route (Peng et al., 2016). As previously reported, phenformin, another biguanide compound, inhibited cell proliferation with IC_50_ 0.57 and 0.25 mM in MB49 and UMUC3, respectively, while the IC_50_ of metformin in these two cell lines were 10.41 and 8.25 mM, respectively (Peng et al., 2016; Huang et al., 2018). These results indicated that phenformin holds 20 to 30 times stronger anticancer capacity than metformin at cellular level (Rajeshkumar et al., 2017). Thus, in this study, we first examined the effect of phenformin on pAkt and pErk1/2. As we expected, the significant inhibition on phosphorylation of both Akt and Erk1/2 was observed in the phenformin group at micromolar concentration (**Figures 4A, B**). Phenformin, 100 µM, has a comparable inhibitory effect to pAkt and pErk1/2 with 4 mM metformin, indicating around 40-fold stronger inhibitory activities. The decrease of the protein levels of pAkt and pErk1/2 was in a dose-dependent manner and was further confirmed with reference to specific inhibitors MK2206 or AZD6244 (**Figure 4C**). Thereby, these results indicated that phenformin may exert dual inhibitory function on both pAkt and pErk1/2, with much stronger efficiency than metformin.

### Synergistic Action of Combination of Phenformin With THP on Proliferation, Colony Formation, and Apoptosis in Bladder Cancer Cells

**Figure 5:**
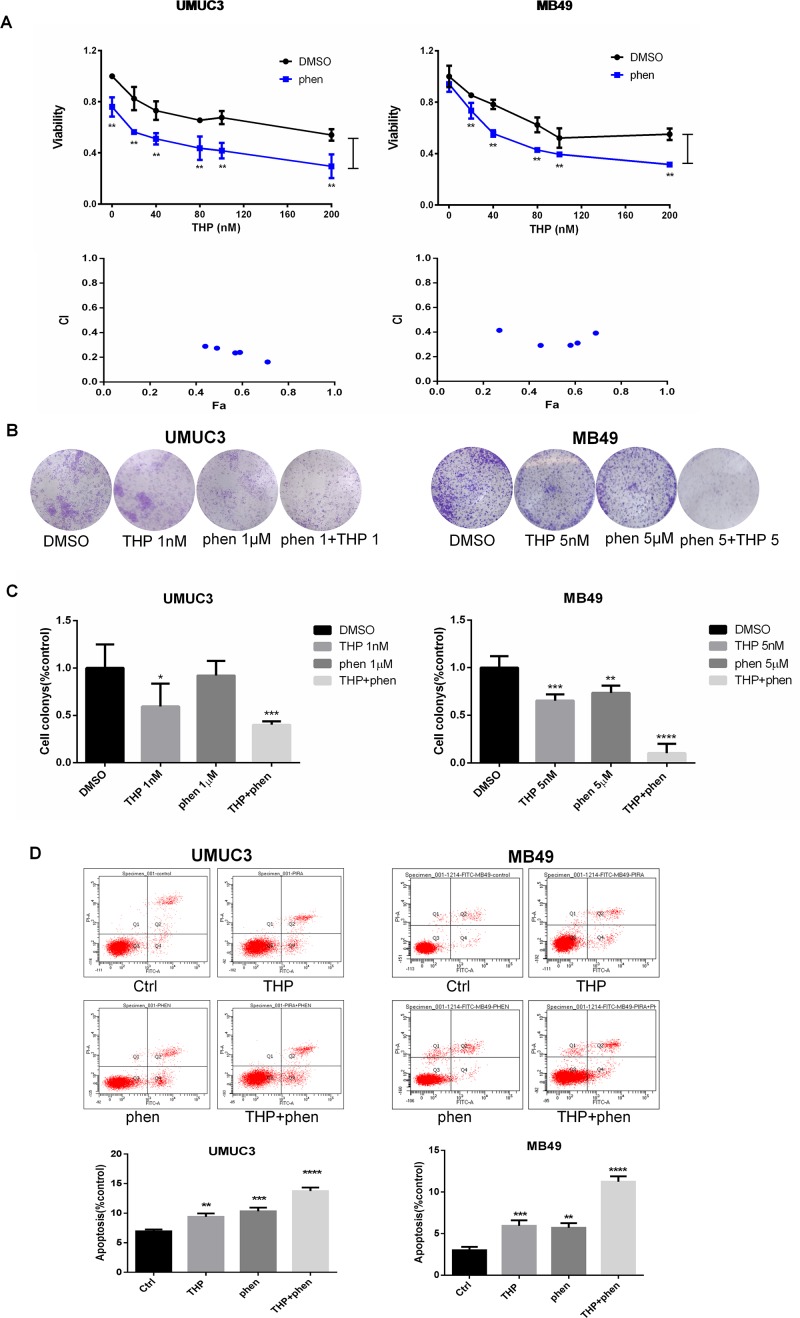
Synergistic action of combination of phenformin with THP on proliferation, colony formation, and apoptosis in bladder cancer cells. **(A)** UMUC3 and MB49 cells were pretreated with 50 µM phenformin for 24 h before exposure to increasing concentrations of THP for an additional 48 h. Cell viability is expressed as a percentage of viability observed in untreated cells. **P < 0.01, *P < 0.05. Below: Combination index (CI) among the combinations of two drugs was calculated using CompuSyn software. If CI = 1, it denotes additives; if CI >1, it denotes antagonism; if CI <1, it denotes synergism. CI values in the vast majority of combinations were 0.2 to 0.5, indicating strong synergism. Results are presented as the median of five independent experiments. **(B)** Colony suppression was evaluated after the treatment of THP combined with phenformin. UMUC3 and MB49 cells were treated for 7 days with THP alone, phenformin alone, or both and then stained with crystal violet to allow colony counting. UMUC3 cells were treated with 1 nM THP, 1 µM phenformin, or both; MB49 cells were treated with 5 nM THP, 5 µM phenformin, or both. **(C)** Quantification of the colonies conducted in panels. Wells were scanned at a wavelength of 550 nm. Results are the mean ± SD of five independent experiments. ***/****P < 0.001, **P < 0.01, *P < 0.05. **(D)** Effect of THP combined with phenformin on apoptosis. UMUC3 and MB49 cells were treated with 200 nM THP alone, 200 µM phenformin alone, or both for 24 h. Representative flow cytometry scatter plots showing propidium iodide (*y* axis) and annexin V–FITC (*x* axis) staining. Quantitation of flow cytometry experiments. Results are the mean ± SD of three independent experiments. ***/****P < 0.001, **P < 0.01, *P < 0.05.

MTT assay was utilized to investigate the antiproliferation effect of combining THP with phenformin. As shown in **Figure 5A**, 50 µM phenformin alone did not significantly inhibit proliferation in MB49 cells, while obvious in UMUC3 cells. Consistent with MK2206 or AZD6244, phenformin exerted profound synergy with THP on inhibiting cell proliferation. The statistical analysis of cell viability between THP and THP + phen groups showed that phenformin dramatically enhanced antiproliferation effect of THP. Interestingly, CI of combining phenformin with THP (CI = 0.2–0.4) is much smaller than the other two combinations of THP with either MK2206 or AZD6244 (0.2–1.0), indicating that combination of phenformin with THP exhibited stronger synergistic effect than combination of THP with single-arm inhibition of either Akt or Erk (**Figure 5A**). We next examined the anticlonogenic effect of combining phenformin with THP. We observed that phenformin significantly amplified the anticlonogenic effect of THP, with increasing colony formation inhibition by 19.3% ± 3.6% in UMUC3 cells, while 12.2% ± 5.2% and 12.7% ± 4.2% with either MK2206 or AZD6244, respectively (**Figures 5B, C**).

Flow cytometry using the annexin V–FITC/PI Apoptosis Kit was used to detect the cell apoptosis. Either THP or phenformin alone substantially increased the proportion of apoptotic cells in UMUC3 and MB49. However, the apoptotic cells were dramatically further increased while combining phenformin with THP (**Figure 5D**).

Taken together, these data demonstrated that dual blocking both Akt and ERK may be a successful strategy to sensitize anticancer activity of THP in bladder cancer.

### Phenformin Alleviated THP-Induced Akt and ERK Phosphorylation and Attenuated THP-Altered mTOR Signaling

**Figure 6:**
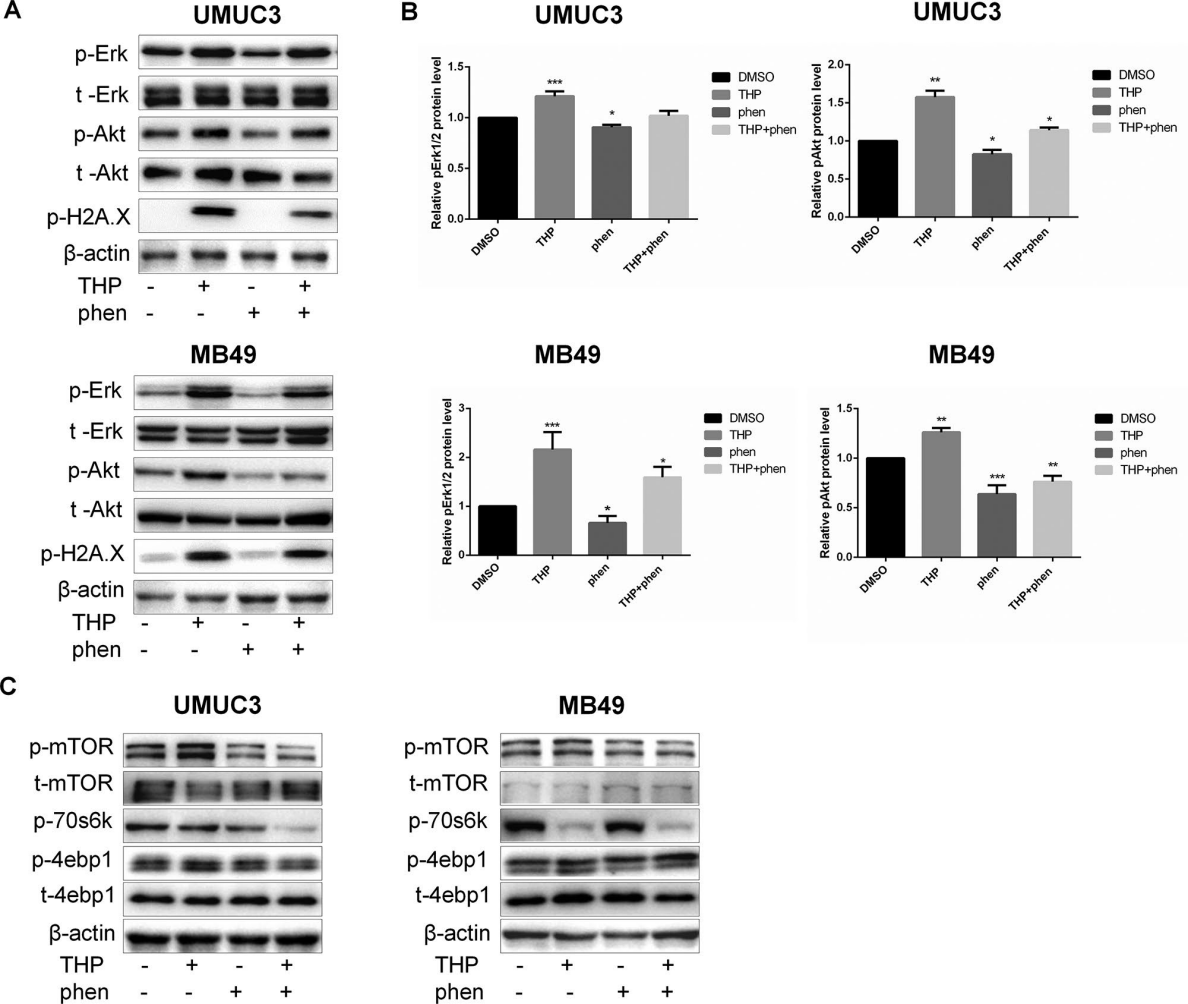
Phenformin alleviated THP-induced Akt and Erk phosphorylation and attenuated THP-altered mTOR signaling. **(A)** UMUC3 and MB49 cells were serum-starved and pretreated with 100 µM phenformin for 2 h before exposure to 400 nM THP for 10 h, and the protein levels of pErk, t-Erk, pAkt, t-Akt, and p-H2A.X were detected by Western blot with β-actin as a control. **(B)** The histogram shows the gray-scale values of the Western blot results. Mean ± SD, n = 3, **/***P < 0.01, *P < 0.05. **(C)** UMUC3 and MB49 cells were serum-starved and pretreated with 100 µM phenformin for 2 h before exposure to 400 nM THP for10 h, and the protein levels of p-mTOR, t-mTOR, p-70s6k, p-4ebp1, and t-4ebp1 were detected by immunoblotting with β-actin as a control.

As shown in **Figure 6A**, THP treatment alone activated Akt and ERK strongly in both human and mice bladder cancer cells. The statistical analysis of protein quantifications in **Figure 6B** verified it (*P* < 0.01). While cells were treated with phenformin alone, both pAkt and pErk1/2 were downregulated, and when cells were pretreated with phenformin before exposure to THP, phenformin could alleviate THP-induced Akt and Erk1/2 phosphorylation (**Figures 6A, B**). On the other hand, previous studies have reported that both Akt and ERK pathways function in parallel to promote mTOR signaling (Winter et al., 2011). Mammalian target of rapamycin is a protein kinase when present in a complex referred to as mTOR complex 1, which acts as an important regulator of cell growth (Laplante and Sabatini, 2013; Wang et al., 2018). Activation of Akt and ERK leads to greater stimulation of mTOR in a synergistic manner (Mendoza et al., 2011). Thus, we aim to detect whether treatment of THP alone or combined with phenformin has any effect on protein expression of mTOR pathway. As shown in **Figure 6C**, THP alone increased the phosphorylation of mTOR and its downstream protein 4EBP1. In contrast, phenformin treatment significantly decreased phosphorylation of these proteins, consistent with our previous observation (Huang et al., 2018). More importantly, phenformin attenuated THP-altered mTOR signaling. Taken together, these results indicated that phenformin reversed THP-induced activation of Akt and ERK and reduced THP-caused mTOR alternations. Interestingly, we discovered that both phenformin and THP significantly decreased the phosphorylation of p70s6k synergistically.

### Synergistic Anticancer Activities of Combining Phenformin With THP *in Vivo*

**Figure 7:**
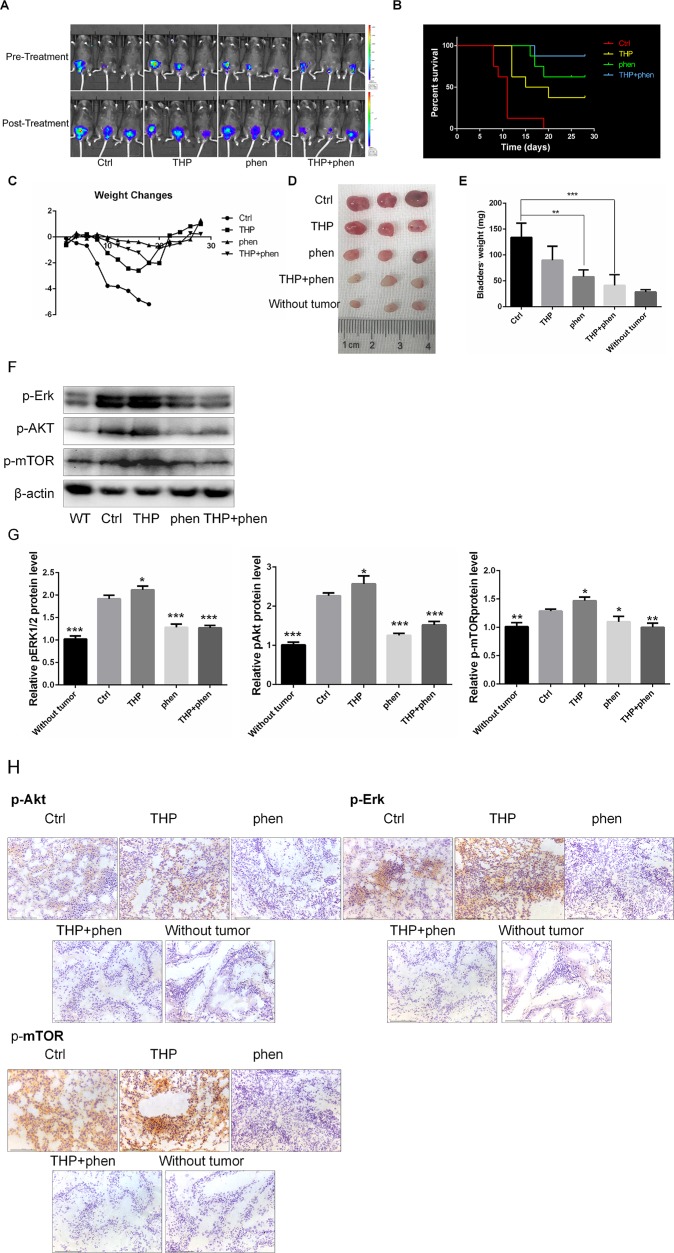
Synergistic action of combining phenformin with THP *in vivo*. **(A)** Images of THP combined with phenformin treated animals in mouse orthotopic implantation model. **(B)** Kaplan–Meier survival analysis of four groups. Death of mice was checked daily, and cumulative survival rate was plotted against the time course. All living mice were killed at day 28. **(C)** The weight of mice was measured daily. All bladder tissues were collected and fixed. **(D)** The images of the explanted bladder tumors. **(E)** Weights of mouse bladders including those that died before the end of experiment were measured. Shown are means and SD. **/***P < 0.01, *P < 00.05. **(F)** Western blot analysis of pAkt, pErk, and p-mTOR protein expression in bladder tissues from mice of five groups. **(G)** Relative levels of phosphorylated Akt, Erk, and mTOR are shown as means ± SD, ***P < 0.001, **P < 0.01, *P < 0.05. **(H)** Immunohistochemistry was used to detect the expression of pAkt, pErk, and p-mTOR of bladder tissues from mice of five groups.

Given the potent synergistic cytotoxic activity of THP with phenformin against bladder cancer cells, we evaluated the antitumor activity *in vivo* using mice bearing MB49^luc^ orthotopic models. The mice implanted with orthotopic bladder tumor were divided into four groups and treated intravesically with 50 µl PBS (Ctrl), THP (0.25mg/ml), phenformin (1.45 mg/ml), and these two drugs’ combinations. All treatments started at day 2 after tumor implantation and continued for 2 weeks. *In Vivo* Imaging System was used to monitor tumor growth. As shown in **Figure 7A**, bladder tumors in mice without drug treatment grew rapidly. THP and phenformin alone decelerated tumor growth, and this deceleration effect was enlarged when combining THP with phenformin. Cumulative survival curves showed cancer cell implantation–induced death of mice (Ctrl), while THP prolonged life span (Ctrl vs. THP, *P* = 0.0019), and the improved efficacy of phenformin on THP was significant (THP vs. THP + phen, *P* = 0.032) (**Figure 7B**). During the period of animal study, total body weight was monitored as evidence for toxicity evaluation. Despite that THP alone suppressed tumor growth, we observed that mice weight in the THP-treated group decreased successively, and mice started to die on the 11th day. In contrast, there is no significant weight loss in the phenformin-treated group. In the group with combined drug treatment, although the weight of these mice decreased slowly, these mice yield the highest survival among these four groups (**Figure 7C**). We dissected the mice and obtained bladders before they were killed. As we can see in **Figure 7D**, bladders in the Ctrl group with instillation of PBS were much bigger than those in any other groups. Bladders in groups with THP or phenformin treated alone were smaller than those in the Ctrl group. The sizes of bladders in the group treated with THP + phenformin were similar to normal bladders, indicating that combination of THP with phenformin achieved the strongest antitumor efficiency among these groups. **Figure 7E** showed that the weights of bladders in the Ctrl group were 134 ± 16.1 mg, while the weights of normal bladders were 28.5 ± 2.2 mg, indicating the attenuation effect of THP or phenformin on tumor-induced burden of bladder weights. The weights of bladders between the group with combination of phenformin and THP and the group without had no significant differences (combination treatment group vs. without, *P* = 0.2726). **Figures 7D and E** further confirmed significant improvement of phenformin on THP efficacy. To detect the changes of the protein levels of bladder tissues in every mouse group, pAkt, pErk1/2, and p-mTOR were measured by Western blot and immunohistochemistry. As shown in **Figures 7F and G**, the expressions of these proteins were increased in the THP-treated group, while decreased in the phen-treated group, compared with these in the Ctrl group. Similar pattern was observed in immunohistochemistry results, shown in **Figure 7H**. These *in vivo* results were consistent with *in vitro* results, confirming that THP activated Akt and ERK, while phenformin significantly downregulated them. In conclusion, these results stated above indicated that phenformin application may be a potential strategy to improve the THP antitumor effect in bladder cancer.

## Discussion

Low response to cytotoxic chemotherapy agents is a common phenomenon in bladder cancer, which influences patient survival (Oosterlinck and Decaestecker, 2018). This underscores the need to identify novel strategies to increase drug response. One approach that has been favored in recent years is the development of drug regimens combining cytotoxic chemotherapy with other drugs, which block the signaling pathway critical to bladder cancer survival and progression (Peng et al., 2017). Non–muscle-invasive bladder cancer is characterized by constitutive activation of the key molecular pathways, including Ras-MAPK and PI3K/Akt pathway (Castillo-Martin et al., 2010; Ouerhani and Elgaaied, 2011). This activation increased patients’ recurrence and progression and decreased their survival (Mitra and Cote, 2009). It has been confirmed that activation of Akt and ERK is crucial for tumor growth and resistance to anticancer drugs (McCubrey et al., 2007; Wang et al., 2009; Liu et al., 2014). In this study, we demonstrated that an undesirable response to THP is the upregulation of both Akt and Erk1/2 phosphorylation, while either Akt or Erk inhibitors dramatically sensitize cells to THP. However, inhibition of one signaling pathway may lead to activation of the other signaling pathway, as we observed the upregulation of either pAkt or pErk1/2 induced by either AZD6244 or MK2206, respectively. These phenomena provide rationale to explore combination therapy of THP and dual pathway inhibitors.

Our previous data showed that phenformin suppressed cell proliferation and migration and induced apoptosis in bladder cancer cells at micromolar concentration, indicating that phenformin has more potent antitumor activity than metformin. The mechanistic investigation showed that phenformin, as metabolism modulator, inhibited cancer growth mainly targeting AMPK and insulinlike growth factor receptor pathways (Guo et al., 2017; Vara-Ciruelos et al., 2019). Here, we found that phenformin significantly decreased the phosphorylation of both Akt and Erk1/2. We further investigated the combinational effects of phenformin with THP. Amazingly, the CI between phenformin and THP was smaller, indicating stronger synergistic efficacy, comparing with the groups of either Akt inhibitor or ERK inhibitor with THP. The further *in vivo* anti–bladder cancer growth assessment confirmed the sensitization of phenformin on THP treatment.

Mammalian target of rapamycin is a serine–threonine protein kinase that is conserved across eukaryotic species. It has been reported that the activation of both Akt and ERK pathways leads to a greater stimulation of mTOR signaling. Our results showed that THP activated mTOR and increased its downstream protein 4EBP1 phosphorylation level. Phenformin sensitized THP accompanied with downregulation of mTOR signaling.

In summary, the present study identifies that two crucial proteins, Akt and Erk1/2, influence insensitivity to THP. And combination of dual inhibitor of Akt and Erk such as phenformin with THP may constitute a successful strategy to improve THP efficacy. However, the detailed mechanisms in Akt, Erk1/2, and mTOR signalings in cell death and apoptosis induced by combination of phenformin with THP deserve further investigations.

## Conclusions

Our study demonstrated that THP treatment significantly activated both Akt and ERK phosphorylation, while phenformin substantially suppressed them. Combination of phenformin as dual inhibitor of Akt and ERK with THP showed strong synergistic anticancer effects. Our findings provide new insights into bladder cancer therapy in the future.

## Data Availability Statement

All datasets generated for this study are included in the manuscript/supplementary files.

## Ethics Statement

The animal study was reviewed and approved by Institutional Animal Care and Use Committee at Hunan Normal University (Protocol 201703230).

## Author Contributions

MP and XY designed this study and drafted the manuscript. JD, SZ, and DX conducted this study and performed the statistical analysis. JL, NZ, CH, and MM prepared the resources.

## Funding

This research was funded by the National Natural Science Foundation of China (81703008, 81874212), Hunan Natural Science Foundation (2018JJ3831, 2016JJ2187), Institutional Fund of Xiangya Hospital Central South University (2016Q02), Xiaoxiang Endowed University Professor Fund of Hunan Normal University (840140–008), and Opening Fund for Key Laboratory of Study and Discovery of Small Targeted Molecules of Hunan Province (2017TP1020).

## Conflict of Interest

The authors declare that the research was conducted in the absence of any commercial or financial relationships that could be construed as a potential conflict of interest.
